# A Novel “Microbial Bait” Technique for Capturing Fe(III)-Reducing Bacteria

**DOI:** 10.3389/fmicb.2020.00330

**Published:** 2020-03-11

**Authors:** Babajide Milton Macaulay, Christopher Boothman, Bart E. van Dongen, Jonathan Richard Lloyd

**Affiliations:** ^1^Department of Earth and Environmental Sciences, The University of Manchester, Manchester, United Kingdom; ^2^Williamson Research Centre for Molecular Environmental Science, The University of Manchester, Manchester, United Kingdom; ^3^Environmental Biology and Public Health Unit, Department of Biology, The Federal University of Technology, Akure, Nigeria

**Keywords:** DIRB, *Geobacter*, *Desulfovibrio*, *Rhodoferax*, pumice, akaganeite, magnetite

## Abstract

Microbial reduction of Fe(III) is a key geochemical process in anoxic environments, controlling the degradation of organics and the mobility of metals and radionuclides. To further understand these processes, it is vital to develop a reliable means of capturing Fe(III)-reducing microorganisms from the field for analysis and lab-based investigations. In this study, a novel method of capturing Fe(III)-reducing bacteria using Fe(III)-coated pumice “*microbe-baits*” was demonstrated. The methodology involved the coating of pumice (approximately diameter 4 to 6 mm) with a bioavailable Fe(III) mineral (akaganeite), and was verified by deployment into a freshwater spring for 2 months. On retrieval, the coated pumice baits were incubated in a series of lab-based microcosms, amended with and without electron donors (lactate and acetate), and incubated at 20°C for 8 weeks. 16S rRNA gene sequencing using the Illumina MiSeq platform showed that the Fe(III)-coated pumice baits, when incubated in the presence of lactate and acetate, enriched for Deltaproteobacteria (relative abundance of 52% of the sequences detected corresponded to *Geobacter* species and 24% to *Desulfovibrio* species). In the absence of added electron donors, Betaproteobacteria were the most abundant class detected, most heavily represented by a close relative to *Rhodoferax ferrireducens* (15% of species detected), that most likely used organic matter sequestered from the spring waters to support Fe(III) reduction. In addition, TEM-EDS analysis of the Fe(III)-coated pumice slurries amended with electron donors revealed that a biogenic Fe(II) mineral, magnetite, was formed at the end of the incubation period. These results demonstrate that Fe(III)-coated pumice “*microbe baits*” can potentially help target metal-reducing bacteria for culture-dependent studies, to further our understanding of the nano-scale microbe-mineral interactions in aquifers.

## Introduction

Microbial interactions with organic matter and minerals drive significant geochemical processes (Lovley, 1997; Vaughan and Lloyd, 2011; Esther et al., 2015). One such process is the interaction of microbes with iron, a redox-active metal dominated by two oxidation states; largely insoluble Fe(III) phases and the more soluble reduced Fe(II) (Vaughan and Lloyd, 2011). In subsurface anaerobic environments, Fe(III) is reduced to Fe(II) by dissimilatory iron-reducing bacteria (DIRB) coupled to the oxidation of organic matter; a process which influences the solubility and mobility of iron and associated metals and metalloids (Lloyd, 2006). DIRB impact sediment minerals in anoxic environments transforming amorphous bioavailable Fe(III) minerals to more crystalline Fe(II) mineral phases (Vaughan and Lloyd, 2011; Esther et al., 2015). Cutting et al. (2009) and Wu et al. (2018), for instance, demonstrated the bioreduction of Fe(III) oxide minerals such as ferrihydrite (Fe_2_O_3_.nH_2_O), akaganeite (β-FeOOH), lepidocrocite (λ-FeO(OH)), feroxhyte (δ-FeO(OH)) and schwertmannite (Fe_8_O_8_(OH)_6_(SO_4_).nH_2_O), to form crystalline iron mineral phases such as siderite (Fe(CO_3_)), vivanite (Fe_3_(PO_4_)_2_.8H_2_O) and magnetite (Fe_3_O_4_). Besides the formation of Fe(II) biominerals, microbial Fe(III) reduction can also play a role in controlling the fate of trace metals and metalloids. DIRB can, for instance, enzymatically reduce soluble U(VI) to insoluble U(IV), limiting radionuclide mobility (Newsome et al., 2018), while Fe(II) associated with biogenic minerals, can reduce and precipitate the radionuclides Tc(VII) and Np(V) to insoluble tetravalent forms (Lloyd et al., 2002; Newsome et al., 2019). Other well-studied immobilization processes for non-radioactive toxic metals include Cr(VI) and Cu(II), reduced by DIRB (and/or the Fe(II) bio-minerals that they form) to insoluble Cr(III) and Cu(I)/(0), respectively (Watts et al., 2015; Kimber et al., 2018). In contrast, DIRB have also been implicated in the mobilization of arsenic (As) in anoxic groundwater. Here, the oxidation of bioavailable organic matter is coupled to the reduction of Fe(III) and As(V) mineral assemblages, resulting in the mobilization of more soluble As(III) (Islam et al., 2004; Cai et al., 2016; Schaefer et al., 2017).

Several studies have reported a wide phylogenetic diversity of bacteria and archaea capable of conserving energy to support growth via dissimilatory Fe(III) reduction (Lovley et al., 1997; Finneran et al., 2003; Lovley, 2006). Bacterial phyla/classes associated with Fe(III) reduction include: Alphaproteobacteria (such as *Azospirillum* sp.); Betaproteobacteria (such as *Rhodoferax ferrireducens* and *Acidovorax* sp.); Deltaproteobacteria (such as *Geobacter* sp., *Pelobacter* sp., *Desulfuromonas acetexigens*, *Desulfovibrio* sp., *Anaeromyxobacter* sp.); Epsilonproteobacteria (such as *Sulfurosprillum* formerly known as *Geospirillum*); Gammaproteobacteria (such as *Shewanella oneidensis*, *S. putrefaciens, S. amazonensis, Wolinella succinogenes*, *Pseudomonas aeruginosa* and *Acinetobacter* sp.); Acidobacteria (such as *Acidobacterium* sp., *Holophaga* sp., *Acidiphilium* sp., *Geothrix fermentans*); Firmicutes (such as *Bacillus* sp., *Clostridium* sp.); and Chloroflexi; Nitrospirae (such as *Nitrospira*); Bacteriodetes; Spirochaetes; Verrucomicrobia; Sphingobacteria (Lovley et al., 1998; Coates et al., 1999; Wilkins et al., 2007; Esther et al., 2015; Hori et al., 2015; Goris and Diekert, 2016; Pan et al., 2017) while Fe(III)-reducing archaea include Euryarchaeota (e.g., *Methanobacterium* sp.) and Crenarchaeota (Pan et al., 2017). The concentration and type of organic matter available was found to control the communities of Fe(III)-reducers (Lentini et al., 2012). For example, the introduction of lactate to series of microcosm experiments enriched for Fe(III)-reducers dominated by *Shewanella, Geothrix, Sulfurospirillum* and *Desulfovibrio* species (Lovley et al., 1997; Lentini et al., 2012; Esther et al., 2015; Stolz et al., 2015; Zhou et al., 2017) while the addition of acetate favored the enrichment of *Geobacter* sp. (Lovley, 1993; Islam et al., 2004; Zhuang et al., 2011; Esther et al., 2015). Interestingly, *Rhodoferax ferrireducens* was found to prefer native organic matter rather than added organic proxies such as acetate and lactate (Zhuang et al., 2011).

Insoluble Fe(III) minerals are utilized as electron acceptors in aquifers, and there is considerable interest in understanding the complex mechanisms of electron transfer to these substrates. The mechanisms are genera-specific and have been studied most intensively in *Geobacter* and *Shewanella* species (Nevin and Lovley, 2002). *Geobacter* species rely on direct contact with *c*-type cytochromes (e.g., *OmcS*), which receive electrons from the menaquinone pool and transfer them to the outer membrane porin *OmpJ* (Leang et al., 2010). Alternatively, electrons can also be carried across proteinaceous nanowires (type IV pili) to external environments (Qian et al., 2011). The conductance of electrons in this arrangement stems from the stacking of aromatic amino acids along the PilA pilin subunit (Vargas et al., 2013). This can extend the transfer of electrons across micrometer distances to the extracellular Fe(III) substrate. Analogous outer membrane *c*-type cytochrome-mediated electron transfer processes are also important in *Shewanella* species. CymA, receives electrons from the menaquinone pool (Myers and Myers, 1997) and transfers them to *MtrA* in the periplasm and then outer membrane cytochromes (OMCs) such as *MtrC*, *MtrF* and *OmcA*, to facilitate extracellular Fe(III) reduction. OMCs in *Shewanella* can also form nano-scale outer membrane and periplasmic extensions analogous to the nanowires noted in *Geobacter* species, but these are not pilin-based structures (Schuetz et al., 2009; Coursolle and Gralnick, 2012; Pirbadian et al., 2014). In addition, *Shewanella* species have been shown to secrete flavins (such as riboflavins and flavin mononucleotide, FMN) which can act as soluble redox-active shuttles, to accelerate Fe(III) reduction (Marsili et al., 2008; von Canstein et al., 2008).

Although many mechanistic laboratory studies have been conducted on a restricted number of *Geobacter* and *Shewanella* species, there remains a need to conduct further targeted experiments under *in situ* conditions using a wider variety of DIRB. To date, the most common means of capturing microbes *in situ* is with the use of “bio-traps” which comprise approximately 50 pieces of BioSep beads (2–3 mm beads engineered with a composite of Nomex and powered activated carbon) (Losi et al., 2006; Taege et al., 2006; North et al., 2012; Xiong et al., 2018). These “bio-traps” are held in Teflon containers equipped with holes and deployed into contaminated groundwater/aquifers for 1–2 months, capturing benzene-degrading microbes (Xiong et al., 2018), ethanol-degrading microbes (North et al., 2012), permanganate-oxidizing bacteria (Taege et al., 2006), chlorinated ethane-reducing bacteria (Losi et al., 2006) and metal/electrode-respiring bacteria (using a graphite electrode as bait) (Badalamenti et al., 2016). However, these “bio-traps” have only been used to deliver organics into the subsurface to enrich for organisms able to degrade pollutants, but have not been used previously (to our knowledge) to target metal-reducing bacteria.

Therefore, the aim of this work was to assess a newly developed bio-trap (thereafter, referred to as a “*microbe bait*”) that could potentially enrich for Fe(III)-reducing communities *in situ*, help identify new organisms able to respire this important extracellular electron acceptor, while also offering a template to study interactions with other organisms in metal-reducing systems (including their interactions with organics that may sorb to Fe(III) mineral phases *in situ*, alongside the nano-scale interactions with iron minerals and other toxic elements). The “*microbe bait*” used here consisted of fragments of pumice (4–6 mm) coated with a poorly crystalline bioavailable Fe(III) oxide mineral phase that could be respired by DIRB in anoxic environments. The objectives of this study were to (1) demonstrate the capture of DIRB using Fe(III)-coated pumice “*microbe baits*” and (2) to compare the impact of natural organic matter captured during deployment to the addition of organic proxies such as lactate and acetate, on the abundance and diversity of the Fe(III)-reducers captured by the “*microbe baits*” from the field, via series of lab-based microcosm experiments. Characterization of the DIRB identified is described, alongside mineralogical and geochemical analyses. In addition, potential future uses of this approach are described.

## Materials and Methods

### Preparation and Identification of Fe(III) Oxide Mineral

An insoluble Fe(III) oxide/oxyhydroxide was prepared following a modified protocol by Schwertmann and Cornell (1991). Ferric chloride hexahydrate (FeCl_3_.6H_2_O; 162 g) was dissolved in 1 L of deionized water and mixed with a magnetic stirrer. The starting pH of the solution was strongly acidic (≤2), and was adjusted to a circumneutral pH by base addition, to precipitate an insoluble Fe(III) mineral. Initially, 200 ml of 10 M NaOH was added to the solution, followed by dropwise additions of 30 ml until the pH was between 6.5 and 7.5, in order to precipitate the iron mineral. The Fe(III) slurry formed was stirred vigorously before centrifuging (Sigma Laboratory Centrifuge 6K15, United States) at 6,000 rpm (4,032 × *g*) for 20 min at 4°C. Dissolved salt was removed from the iron slurry by washing with deionized water four times. The mineralogy of the iron slurry was assessed by (powder) X-ray diffraction (XRD) using a *Bruker D8 Advance* (United States), operating at 40kv/40mA, with Cu-Kα1 radiation (λ = 1.5418), and was stored at 4°C for future use.

### Coating of Pumice With the Fe(III) Mineral

Pumice samples, of approximately 4 to 6 mm diameter, were purchased from Fisher Scientific Ltd., United Kingdom. To remove impurities (e.g., dust and debris) to open up the pores of the pumice, and increase the surface area for adsorption (Kumar et al., 2008; Far et al., 2012) the pumice was suspended in 37% HCl for 24 h and washed repeatedly until the run off was clear of particles and acid. The protocol of Tufano et al. (2008) was followed for coating the pumice with the Fe(III) mineral. The acid-washed pumice (10 g) was mixed thoroughly with 10 ml of the prepared iron slurry and allowed to air-dry for 2 days. Excess slurry was removed with 200 ml deionized water, the Fe(III)-coated pumice was allowed to air dry for 3 days and stored dry for further use. Supplementary Figures S1A,B shows the comparative appearance of the uncoated and Fe(III)-coated pumice samples. Prior to deployment, pumice samples (unwashed, acid-washed, uncoated, Fe(III)-coated and thin-sectioned) were characterized using a Quanta FEG 650 Environmental Scanning Electron Microscope (ESEM) (*FEI Instrument*, United States) fitted with an Energy Dispersive x-ray Spectroscopy (EDS) unit (*Bruker x Flash 6130 Instrument* (United States), Esprit 2.1 software). The ESEM was set at high voltage (15 kv), with a spot size of 3.50 and a chamber pressure of 0.30 mbar. A scanning speed of 3 μs was used, with a working distance of 8.9–9.2 mm, and a concentric-backscatter (CBS) detector.

### Deployment in the Field

**FIGURE 1:**
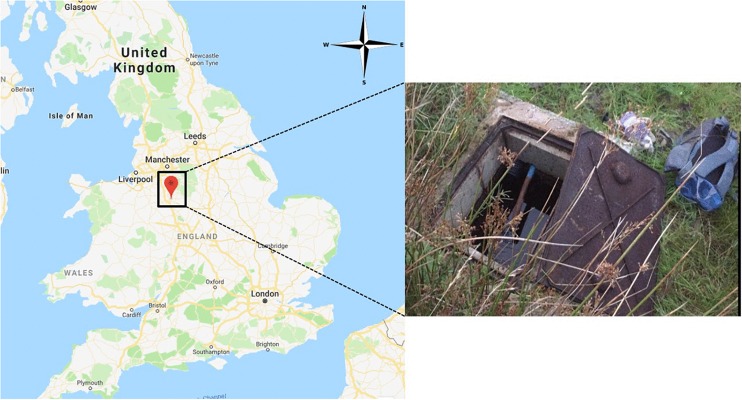
Spring water sampling site at a private spring in the West Midlands, United Kingdom.

Uncoated and Fe(III)-coated pumice samples (10 g) were held in sample bags constructed from nylon stockings (Supplementary Figure S1C) submerged (30–40 cm depth) in a private drinking water well built over a spring in the West Midlands, United Kingdom (Figure 1). Pumice samples were deployed in the well for 2 months, retrieved in air-tight acid-washed containers filled with spring water, and returned to the laboratory at The University of Manchester for further analysis.

### Laboratory Incubations and Analyses

On arrival at the University of Manchester, the retrieved uncoated and Fe(III)-coated pumice samples were removed from the nylon sample bags in an anaerobic chamber and were placed into autoclaved serum bottles. Sterile synthetic groundwater (SGW) was prepared following the modified method of Wilkins et al. (2007), which contains (in grams per liter of deionized water): KCl (0.0066); MgCl_2_.6H_2_O (0.081); CaCO_3_ (0.1672); sodium silicate (0.0829); NaNO_3_ (0.0275); NaCl (0.0094); NaHCO_3_ (0.2424). MgSO_4_.7H_2_O was excluded from the SGW recipe as it could encourage the enrichment of sulfate-reducing bacteria over iron-reducing bacteria. The pH of the SGW (8.5) was reduced to 7.1 with CO_2_, and 40 ml aliquot was added into each 100 ml serum bottle containing 10 g pumice, i.e., artificial groundwater to sediment ratio of 4:1. Three sets of treatment were set-up: (1) Uncoated versus Fe(III)-coated pumice samples retrieved from the field but not amended with carbon source. (2) Uncoated versus Fe(III)-coated pumice samples retrieved from the field but amended with 15 mM (∼1,600 mg/L) sodium lactate and 15 mM (∼1,600 mg/L) sodium acetate (added directly into the synthetic groundwater medium). (3) Uncoated versus Fe(III)-coated pumice samples not deployed in the field and unamended with electron donors (which served as the negative control). The headspace of the serum bottles was flushed with nitrogen (to create anoxic conditions similar to field scenarios), sealed with butyl rubber stoppers and incubated in the dark at 20°C for 8 weeks (56 days). All three batches of experiments were set up in triplicate (Supplementary Figure S2A). Due to the cost involved in analyzing for the microbial community in the Springwater and coated/uncoated pumice samples, a single subsample was collected and analyzed for the Springwater sample. As for the coated/uncoated pumice samples freshly retrieved from the field, duplicate subsamples were collected and analyzed at the end of the microcosm experiment. Duplicate samples with the highest degree of iron transformation (i.e., color change from brick red to black) were preferentially selected.

Prior to the microbial incubation experiment, geochemical screening of the spring water (Table 1) as well as the X-ray fluorescence (XRF) spectrometry (*Axios, PANalytical, Almelo, The Netherlands*) and XRD characterization of the pumice samples (prior to deployment in the field) were carried out. For the XRF, concentrations of major elements such as Fe and Si, were determined on air-dried, finely-ground and pressed powdered briquettes, composed of 6.7 g pumice and 1.7 g wax (Table 2).

**TABLE 1:** Bulk geochemical analyses of the spring water sample collected in June 2017. Data are means ± standard error values.

Analytes	Concentrations (mg/L)
Fe	0.14 (±0.01)
Ca	7.12 (±0.03)
Mg	2.15 (±0.00)
K	0.96 (±0.01)
Mn	0.58 (±0.01)
P	0.01 (±0.03)
S	5.89 (±0.14)
Na	3.63 (±0.22)
Al	0.22 (±0.00)
Mo	0.01 (±0.01)
Chloride	8.65 (±0.03)
Sulfate	15.51 (±0.31)
Nitrate	8.72 (±0.28)
Dissolved organic carbon (DOC)	1.55 (±0.88)
Carbonate	0.00 (±0.00)
Bicarbonate	3.47 (±0.17)

**TABLE 2:** Major element concentrations of ferric oxide (Fe_2_O_3_) and silica oxide (SiO_2_) in pumice samples (in wt%); Data are means ± standard error values.

Sample	Fe_2_O_3_	SiO_2_
Uncoated pumice	1.58 (±0.02)	72.82 (±0.14)
Fe(III)-coated pumice	3.15 (±0.28)	72.00 (±0.21)

Pumice samples from the laboratory incubations were collected once a week for a series of physicochemical analyses. These included determination of the pH and redox potential (*Eh*) (analyzed with a calibrated pH-*Eh* Denver instrument meter), and dissolved organic carbon (DOC) content by centrifuging a 1 ml aliquot of the pumice slurry in a Sigma 1-14 Centrifuge at 14,800 rpm (24,532 × *g*) for 5 min, and analyzing the supernatant using a Total Organic Carbon (TOC) Analyzer (Shimadzu TOC-V CPN, Japan). The production of Fe(II) in the pumice slurry was measured using the ferrozine assay as described by Lovley and Phillips (1988). Cations/metals were analyzed using inductively coupled plasma-atomic emission spectroscopy, ICP-AES (Optima 5300DV; PerkinElmer, United States), with detection limits of 10–15 μg/L while anions and organic acids were measured using an ion chromatography (IC) system (Dionex ICS5000, United States) with a dual channel system comprising microbore and capillary channels, with detection limits of 0.05 and 0.01 mg/L, respectively.

Mineralogical changes in the pumice slurry (at the end of the experiment) were assessed using ESEM and Transmission Electron Microscope (TEM). The TEM used was a Philips/FEI CM200 model (United States), equipped with a Field Emission Gun, Energy Dispersive x-ray Spectroscopy (EDS) unit (Oxford Instruments UTW ISIS, United Kingdom) and a Gatan Imaging Filter. All TEM images were produced using an operating beam voltage of 200 keV. Elemental measurements of the samples were obtained using EDS. Selected area electron diffraction (SAED) patterns were also acquired using appropriate diffraction apertures, to help identify any new minerals that were formed at the end of the experiment. Prior to imaging, a droplet of each pumice slurry sample was placed on a carbon 300 mesh copper grid (Agar Scientific, United Kingdom) and allowed to air-dry. The ESEM was set at variable pressure mode under low vacuum. The chamber pressure was 0.70 m bar while other parameters were set to conditions previously described.

### Microbial Community Analysis

#### DNA Extraction

DNA was extracted from 200 μl of enrichment medium or 0.2 g of uncoated and Fe(III)-coated pumice sample (retrieved from field) and pumice slurries (after incubation) using a DNeasy PowerLyzer PowerSoil Kit (Qiagen, Manchester, United Kingdom). In addition, 100 ml of the spring water sample was filtered through 47 mm Whatman sterile membrane filters to concentrate the biomass; DNA was extracted from the biomass using the DNeasy PowerWater Kit (Qiagen, Manchester, United Kingdom). The 16S rRNA gene was amplified via PCR (polymerase chain reaction) using 8F (5′-AGAG TTTGATCCTGGCTCAG-3′), and 1492R (5′-TACGGYTACCT TGTTACGACTT-3′) primers using the methods of Rizoulis et al. (2016). The primer pair 8F-1492R was purely used to check that the DNA extraction procedure had isolated DNA containing the bacterial 16S rRNA gene, prior to sequencing reactions being carried out on the samples. Following amplification via PCR, the DNA was stained before placement in an agarose gel, where it was subsequently separated using electrophoresis. The stained DNA was viewed under UV light, and target ∼1500 base pair products were identified by comparison to a ladder of DNA fragments of varying lengths.

#### Sequencing

Sequencing of PCR amplicons of 16S rRNA genes was conducted with the Illumina MiSeq platform (Illumina, San Diego, CA, United States) targeting the V4 hyper variable region (forward primer, 515F, 5′-GTGYCAGCMGCCGCGGTAA-3′; reverse primer, 806R, 5′-GGACTACHVGGGTWTCTAAT-3′) for 2 × 250-bp paired-end sequencing (Illumina) (Caporaso et al., 2011, 2012). PCR amplification was performed using Roche FastStart High Fidelity PCR System (Roche Diagnostics Ltd., Burgess Hill, United Kingdom) in 50 μl reactions under the following conditions: initial denaturation at 95°C for 2 min, followed by 36 cycles of 95°C for 30 s, 55°C for 30 s, 72°C for 1 min, and a final extension step of 5 min at 72°C. The PCR products were purified and normalized to ∼20 ng each using the SequalPrep Normalization Kit (Fisher Scientific, Loughborough, United Kingdom). The PCR amplicons from all samples were pooled in equimolar ratios. The run was performed using a 4pM sample library spiked with 4pM PhiX to a final concentration of 10% following the method of Kozich et al. (2013).

Raw sequences were divided into samples by barcodes (up to one mismatch was permitted) using a sequencing pipeline. Quality control and trimming was performed using Cutadapt (Martin, 2011), FastQC (Babraham Bioinformatics, 2018), and Sickle (Joshi and Fass, 2011). MiSeq error correction was performed using SPADes (Nurk et al., 2013). Forward and reverse reads were incorporated into full-length sequences with Pandaseq (Masella et al., 2012). Chimeras were removed using ChimeraSlayer (Haas et al., 2011), and Operational Taxonomic Units (OTUs) were generated with UPARSE (Edgar, 2013). OTUs were classified by Usearch (Edgar, 2010) at the 97% similarity level, and singletons were removed. Rarefaction analysis was conducted using the original detected OTUs in Qiime (Caporaso et al., 2010). The taxonomic assignment was performed by the RDP classifier (Wang et al., 2007). Although the RDP Classifier only provides genus level confirmation but in order to determine species level identity, the nucleotide sequence of the OTU‘s were also analyzed against the NCBI Blast database.

## Results

### Characterization of the Fe(III) Oxide Mineral Prepared and Coated Pumice Samples Prior to Deployment

**FIGURE 2:**
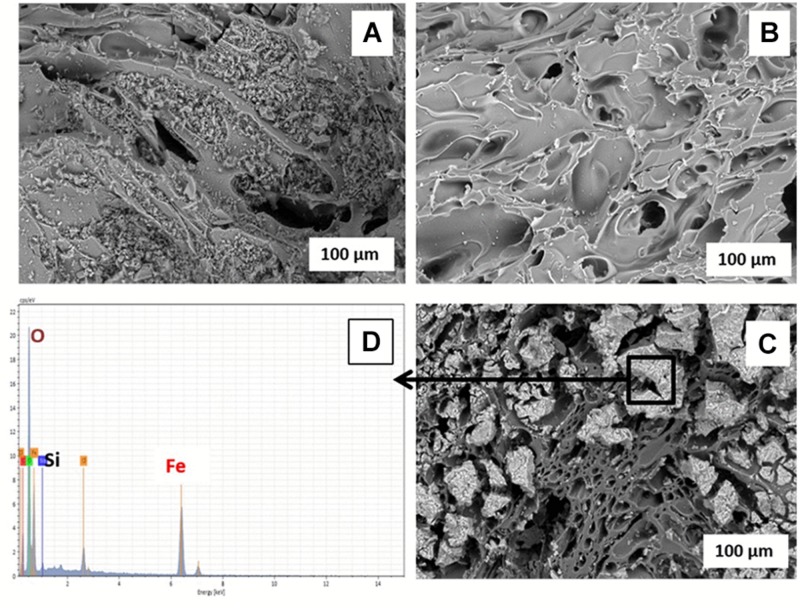
ESEM micrographs of **(A)** unwashed uncoated pumice, **(B)** acid-washed uncoated pumice, **(C)** akaganeite (Fe(III)-coated pumice, and **(D)** EDS spectrum of Akaganeite-coated pumice.

The Fe(III) oxide mineral prepared and coated on the pumice was identified by XRD as akaganeite (β-FeOOH; Supplementary Figure S3). Comparative analysis of the ESEM micrographs (Figures 2A,B) shows the significance of soaking the pumice in 97% HCl for 24 h prior to the coating process, as recommended by Kumar et al. (2008) and Far et al. (2012). The acid-washing process clearly helped to remove small pumice particles blocking the pores on the surface and inside the uncoated pumice, the presence of which could potentially reduce the surface area for Fe(III) adsorption. The Fe(III)-coated pumice samples showed the presence of large particles attached to the surface of the pumice which were identified as Fe minerals by EDS (Figures 2C,D). In addition, thin-sections of the coated pumice examined under the ESEM-EDS revealed that the Fe(III) oxide mineral penetrated into the interior pores and crevices of the pumice as well (Supplementary Figure S4).

### Geochemical Evidence for Fe(III) Reduction in the Microbial Incubation Experiments

#### Changes in Fe(II) Concentrations, Dissolved Organic Carbon (DOC), pH and Redox Potential (*Eh*)

Uncoated/Fe(III)-coated pumice samples retrieved from the spring water well, unamended and amended with electron donors, were incubated anaerobically for 8 weeks and analyzed for pH, *Eh*, DOC and evidence of Fe(II) release.

**FIGURE 3:**
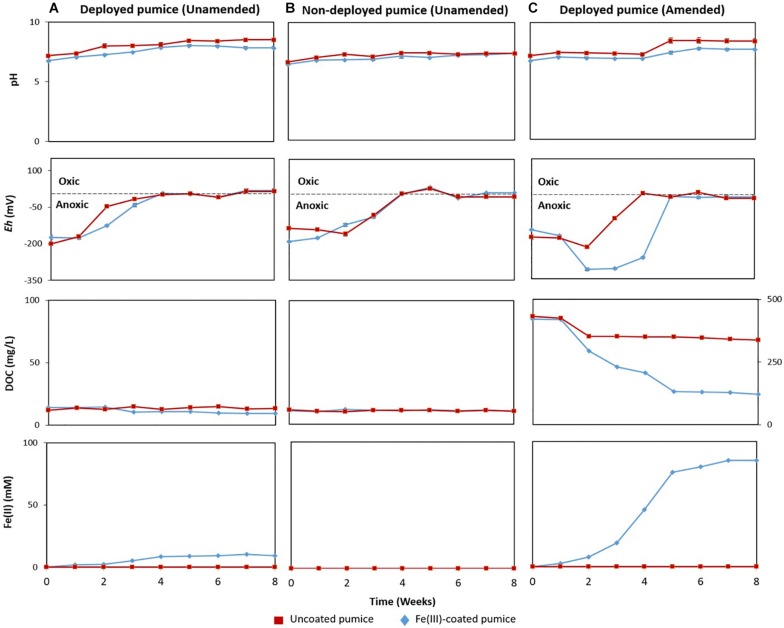
Measurements of pH, redox potential (*Eh*), dissolved organic carbon (DOC) and Fe(II) concentrations, in anaerobic microcosms of pumice slurries incubated at 20°C for 8 weeks and set up in the following batches: **(A)** Deployed pumice unamended with electron donors, **(B)** Non-deployed pumice unamended with electron donors. **(C)** Deployed pumice amended with lactate and acetate. Data are means ± standard errors of triplicate.

In the deployed Fe(III)-coated pumice slurries unamended with electron donors, Fe(II) concentrations increased to 9 mM at week 4 and remained stable until the end of the incubation period, consistent with minor depletion of DOC from 13.9 to 9.5 mg/L (Figure 3A). Deployed uncoated pumice slurries unamended with electron donors showed no sign of Fe(II) release. The addition of lactate and acetate to deployed Fe(III)-coated pumice slurries, led to a much higher increase in the concentration of Fe(II), reaching up to 85 mM; consistent with DOC depletion from 421 to 120 mg/L (Figure 3C). As expected, non-deployed uncoated/Fe(III)-coated pumice slurries unamended with electron donors showed no sign of Fe(III) reduction nor DOC depletion (Figure 3B). In all the pumice treatments, pH values were generally circumneutral (between 6.5 and 8.5), but trended upward slightly (7.4 to 8.4) in the deployed Fe(III)-coated pumice slurries amended with electron donors at week 4, which corresponded with significant Fe(III) reduction (Figure 3). In all the pumice treatments, the *Eh* values initially indicated a reductive condition (approximately −200 mv) but increased to near zero values (oxic) by the end of the incubation period. However, in the deployed Fe(III)-coated pumice slurries amended with electron donors, the *Eh* was further reduced from approximately −200 to −330 mv, between weeks 2 and 5, before increasing to near zero by the end of the experiment (week 8).

#### Organic Matter Oxidation

**FIGURE 4:**
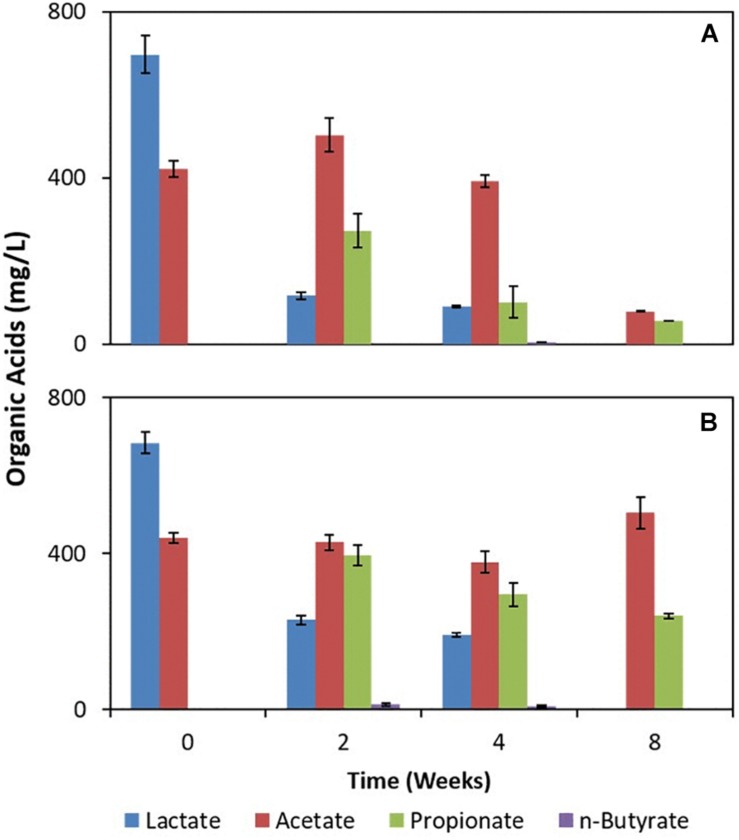
Measurements of organic acids in pumice slurries amended with electron donors and incubated at 20°C over the period of 8 weeks. **(A)** Fe(III)-coated sample deployed and amended with lactate and acetate. **(B)** Uncoated pumice sample deployed and amended with lactate and acetate. The data are the means ± standard errors of triplicates.

The deployed uncoated/Fe(III)-coated pumice slurries amended with lactate and acetate showed incomplete oxidation of lactate to propionate and n-butyrate (Figure 4). However, acetate was largely fully oxidized in the deployed Fe(III)-coated pumice slurries compared to the deployed uncoated pumice slurries which appeared relatively stable. Furthermore, there was a more aggressive consumption of organic acids in the deployed Fe(III)-coated pumice slurries compared to the deployed uncoated pumice slurries. For instance, the total organic acids in the deployed Fe(III)-coated pumice slurries at week 0 was ∼1,100 mg/L (comprising lactate and acetate) and was biodegraded to 130 mg/L (comprising acetate and propionate) by the end of the experiment compared to the total organic acids in the deployed uncoated pumice slurries which was poorly biodegraded to ∼700 mg/L (comprising acetate and propionate). As expected, organic acids were not detected in the non-deployed uncoated/Fe(III)-coated pumice slurries unamended with electron donors (not shown).

#### The Fate of Sulfate and Nitrate During Fe(III) Reduction

**FIGURE 5:**
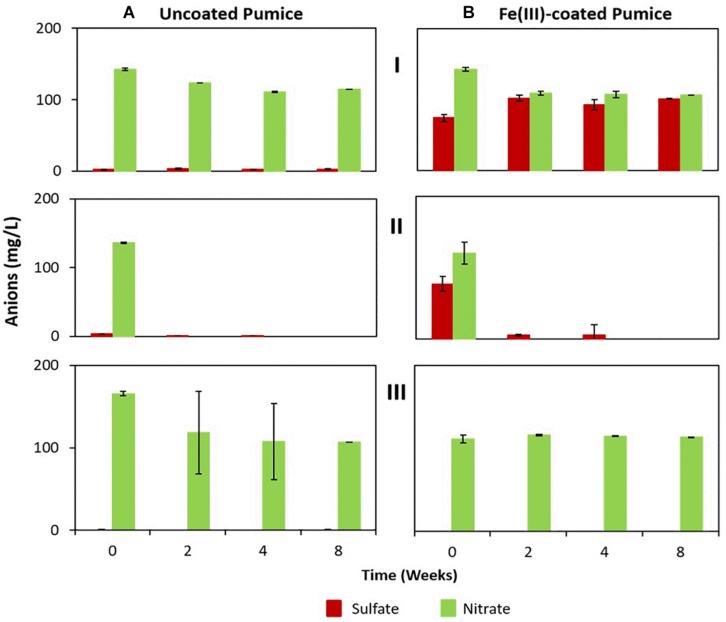
Sulfate and nitrate concentrations in the deployed uncoated/Fe(III)-coated pumice slurries incubated for 8 weeks at 20°C. (I) Deployed pumice unamended with electron donors. (II) Deployed pumice amended with lactate and acetate. (III) Non-deployed pumice unamended with electron donors (Control). Data are the means ± standard errors of triplicates. **(A)** Uncoated pumice treatment. **(B)** Fe(III)-coated pumice treatment.

Although sulfate and nitrate were not added to the microcosm enrichments they were detected in the deployed pumice slurries (Figure 5). Deployed uncoated pumice slurries unamended/amended with electron donors had low concentrations of dissolved sulfate (2.2 to 3.4 mg/L), while the non-deployed uncoated pumice slurries had lower amounts of dissolved sulfate (0.5 to 0.8 mg/L). However, higher concentrations of sulfate (74 to 101 mg/L) were detected in the deployed Fe(III)-coated pumice slurries (both unamended and amended with lactate and acetate), while the non-deployed Fe(III)-coated slurries had much lower sulfate concentrations (0.2 to 0.5 mg/L) similar to the slurries containing uncoated pumice. However, the concentrations of nitrate detected in the deployed uncoated/Fe(III)-coated pumice slurries unamended/amended (120 to 142 mg/L) were similar to those detected in the non-deployed uncoated/Fe(III)-coated pumice slurries unamended/amended (111 to 118 mg/L).

In the deployed uncoated/Fe(III)-coated pumice slurries unamended with electron donors, there was no observable changes in the concentrations of sulfate and nitrate over the whole incubation period. In contrast, the sulfate and nitrate in the deployed but uncoated/Fe(III)-coated pumice slurries, amended with electron donors, were completely reduced by the end of the experiment. Sulfate reduction in the deployed Fe(III)-coated pumice slurries amended with electron donors occurred concurrently with DOC depletion and Fe(II) release (Figure 3C). Similarly, total sulfur concentrations in the aqueous phase reduced from 10 mg/L to 0.3 mg/L at the end of the incubation period (Supplementary Figure S5). Sulfur was however, detected in high concentrations in the aqueous phase in the deployed Fe(III)-coated pumice slurries unamended with electron donors (Supplementary Figure S5).

### Identification of the Biogenic Fe(II) Mineral Formed During the Microbial Incubation Experiments

**FIGURE 6:**
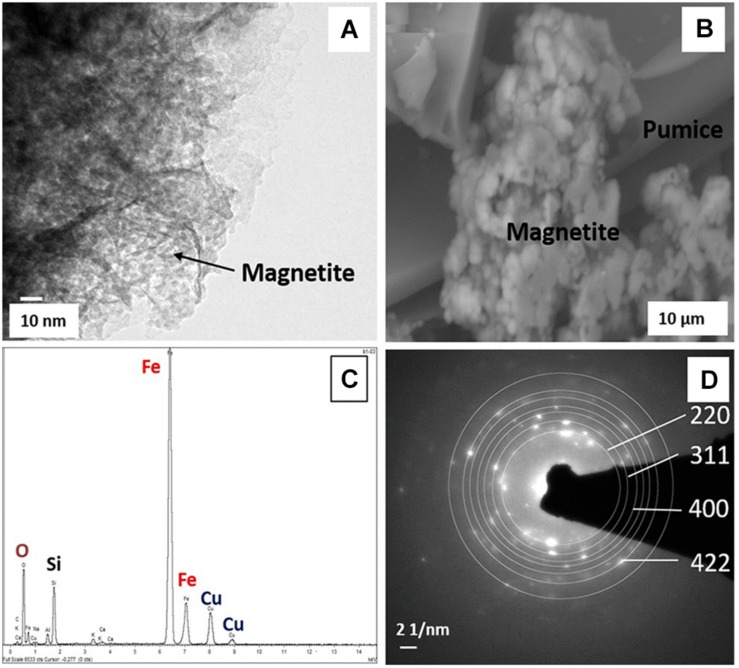
**(A)** TEM and **(B)** ESEM micrographs, **(C)** EDS spectrum, and **(D)** SAED patterns of biogenic magnetite obtained from the deployed Fe(III)-coated pumice slurries amended with lactate and acetate, incubated for 8 weeks at 20°C.

Only the deployed Fe(III)-coated pumice slurries amended with electron donors showed noticeable color changes, turning from brick-red to black, at the end of the experiment (Supplementary Figure S2). TEM and ESEM analyses of the black-colored Fe(II) mineral formed from the lactate/acetate-stimulated bioreduction of akaganeite during the microbial incubation experiment were carried out. TEM analyses of the black-colored Fe(II) mineral formed shows spherical nanoparticles with an average diameter of 6 nm (Figure 6A) lying on the pumice (Figure 6B). Furthermore, the nanocrystals appeared as aggregates of roughly spherical structures lying on the pumice surface. EDS analyses revealed the presence of Fe, O, Si, and Cu (Figure 6C) and analysis of the SAED patterns using the *Image J* software revealed that the diffraction rings (220, 311, 400 and 422) corresponded with the following *d* values: 2.99, 2.60, 2.13, and 1.80 A, respectively (Figure 6D).

### Fe(III)-Reducers Captured by the Deployed “Microbe Baits”

**FIGURE 7:**
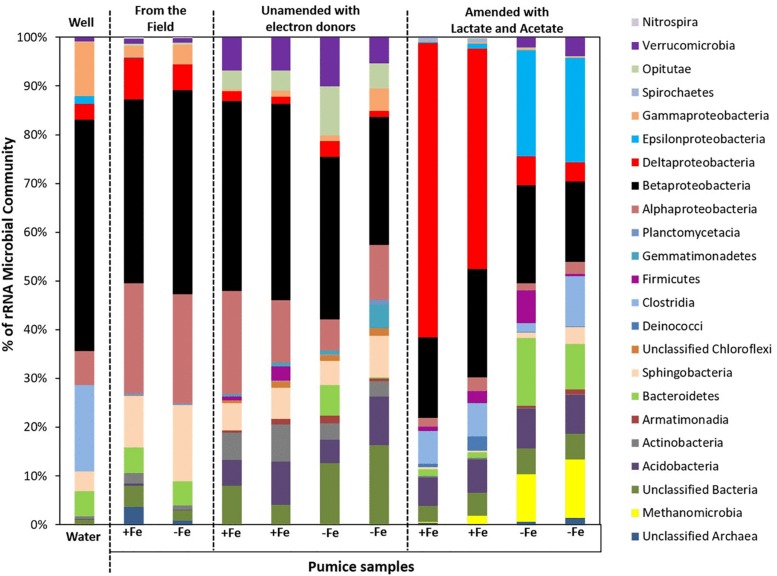
Microbial community (Phyla/classes) in spring water and deployed pumice samples incubated (with and without electron donors) for 8 weeks at 20°C. +Fe represents Fe(III)-coated pumice and –Fe depicts uncoated pumice. Duplicates of each pumice sample were analyzed.

Analyses (using high throughput Illumina 16S rRNA gene sequencing) of spring water samples and the microorganisms captured by freshly retrieved uncoated and Fe(III)-coated pumice that had been deployed into the spring water well for 2 months were carried out. The retrieved Fe(III)-coated and uncoated pumice captured the following microbial Phyla/Classes from the spring water: Betaproteobacteria (38 and 42%), Alphaproteobacteria (23 and 22%), Sphingobacteria (11 and 16%), Deltaproteobacteria (9 and 5%), Bacteriodetes (5% each), Gammaproteobacteria (2 and 4%), Verrucomicrobia (1% each) and Clostridia (0.03 and 0.05%; Figure 7). The spring water sample also contained similar microbial groups: 47% Betaproteobacteria, 18% Clostridia, 11% Gammaproteobacteria, 7% Alphaproteobacteria, 5% Bacteriodetes, 4% Sphingobacteria, 3% Deltaproteobacteria, 2% Epsilonproteobacteria, 1% Verrucomicrobia, and 2% others. At the end of anoxic incubation, the deployed Fe(III)-coated pumice slurries unamended with electron donors enriched a higher abundance of microbes than the deployed uncoated pumice slurries unamended with electron donors: Betaproteobacteria (40 and 19%), Alphaproteobacteria (17 and 6%), Sphingobacteria (6 and 5%), Actinobacteria (7 and 2%), Verrucomicrobia (7 and 5%), Opitutae (4 and 5%), Acidobacteria (7 and 5%), Deltaproteobacteria (2% each), and Gammaproteobacteria (1 and 2%). The addition of electron donors during laboratory incubations altered the composition of the microbial community. Deltaproteobacteria (53%) dominated in the deployed Fe(III)-coated pumice slurries amended with electron donors. Interestingly, deployed uncoated pumice slurries amended with electron donors preferentially enriched Epsilonproteobacteria (22%) and Methanomicrobia (11%).

**FIGURE 8:**
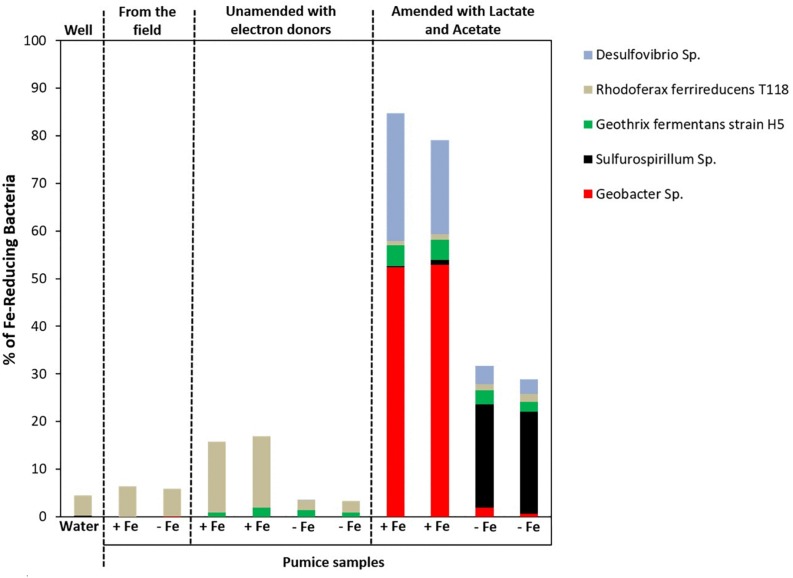
Microbial community (Genera) in spring water and deployed pumice samples incubated (with and without electron donors) for 8 weeks at 20°C. +Fe represents Fe(III)-coated pumice and –Fe depicts uncoated pumice. Duplicates of each pumice sample were analyzed.

Microbial community analysis at a greater taxonomic resolution showed that both the freshly retrieved and incubated Fe(III)-coated and uncoated pumice slurries unamended with electron donors enriched for a close relative to *Rhodoferax ferrireducens* (6.4 and 5.8%, freshly retrieved; 15 and 2%, incubated with no electron donor; Figure 8). Similar to previous observations (Islam et al., 2004; Rowland et al., 2007; Hery et al., 2010; Zhuang et al., 2011; Farkas et al., 2017), electron donor addition altered the community diversity at the Genus level. Deployed Fe(III)-coated pumice slurries amended with electron donors enriched a higher population of Fe(III)-reducers (80% of the identified species) compared to the deployed uncoated pumice slurries amended with electron donors (31% of the identified species). For example, *Geobacter* sp. (52%) and *Desulfovibrio* sp. (24%) were largely enriched in the deployed Fe(III)-coated pumice slurries amended with electron donors, which is consistent with observations at the Phyla/Class level (Deltaproteobacteria, 53%) for the same sample. In the deployed uncoated pumice slurries amended with electron donors, *Sulfurospirillum* species (22%), belonging to class Epsilonproteobacteria, were the most abundant microbial species identified; which is consistent with the abundance of Epsilonproteobacteria (22%) at the Phyla/Classes level.

## Discussion

### Geochemical Changes During the Incubation of the Deployed “Microbe Baits”

The XRD pattern of the akaganeite mineral prepared (Supplementary Figure S3) is consistent with the patterns reported by Lee et al. (2003) and Cutting et al. (2009). Akaganeite is bioreducible and has been reported to be reduced to magnetite by Fe(III)-reducing bacteria (Lee et al., 2003; Cutting et al., 2009); therefore, is an appropriate substrate to select for the capture and enrichment of Fe(III)-reducing bacteria *in situ*.

There was a minor level of Fe(III) reduction in the deployed Fe(III)-coated pumice slurries unamended with electron donors (9 mmol Fe(II) per liter coated pumice slurry), compared to the Fe(III)-coated pumice slurries amended with electron donors (85 mmol Fe(II) per liter coated pumice slurry). This implies that low levels of electron donor used for Fe(III) reduction (consistent with DOC depletion from 13.9 to 9.5 mg/L) was captured by the Fe(III)-coated pumice during deployment in the field, despite the relatively low levels of DOC in the spring water (as low as ∼2 mg/L; Table 1). In all three pumice slurry samples, the *Eh* generally indicated a highly reductive condition (−200/−330 mv) and increased to near zero values at the end of the incubation period. The incubations containing deployed Fe(III)-coated pumice slurries amended with electron donors, had the lowest *Eh* value (−330 mv) close to values associated with sulfate-reducing conditions (Christian et al., 2010; Gorny et al., 2015), indicating that sulfate-reducers could also be contributing to the Fe(III)-reduction.

Sulfate reduction leads to sulfide formation, which reduces Fe(III) to Fe(II) abiotically, forming FeS (Coleman et al., 1993; Wang et al., 2017) while nitrate reduction initiates Fe(II) oxidation via the formation of reactive nitrite species (Sorensen, 1982; Smith et al., 2017). Both processes can be coupled to the oxidation of organic matter (Weber et al., 2006). Sulfate reduction in the deployed Fe(III)-coated pumice slurries amended with electron donors occurred concurrently with DOC depletion and Fe(II) release (Figure 3C), but its contribution to Fe(III) reduction was minor compared to that of Fe(III)-reducers. This is because the Fe(III) coating on the pumice would encourage the activities of Fe(III)-reducers over that of sulfate-reducers. The stable concentrations of sulfate and nitrate observed in the deployed uncoated/Fe(III)-coated pumice slurries unamended with electron donors may be a result of the insufficient amount of organic matter captured by the pumice to support bioreduction of the nitrate and sulfate. However, the biostimulation of the deployed uncoated/Fe(III)-coated pumice slurries amended with election donors accelerated the reduction of nitrate and sulfate, in line with previous studies (Smith et al., 2017; Wang et al., 2017).

In the deployed uncoated/Fe(III)-coated pumice slurries amended with electron donors, the lactate and acetate was partially and completely oxidized, respectively. This indicates that the akaganeite coating on the pumice may have led to the enrichment of a diverse distribution of Fe(III)-reducers capable of oxidizing both lactate and acetate in anoxic conditions. As for the deployed uncoated pumice slurries, lactate metabolism was observed in the absence of an electron acceptor (Fe(III); Figure 4B), suggesting the presence of other electron acceptors, such as insoluble manganese or sulfate minerals (Sousa et al., 2017; Novotnik et al., 2019), sorbed or precipitated from the spring water. Alternatively, fermentation of lactate could also occur in the absence of an electron acceptor (Oude-Elferink et al., 2001; Tao et al., 2016). This indicates that the source of the sulfate in the deployed Fe(III)-coated pumice slurries is most likely from the spring water well. Oxoanions such as sulfate can potentially be adsorbed and accumulated onto the surface of Fe(III) minerals (Hinkle et al., 2015) and could co-exist to enhance sequential Fe(III)-sulfate reduction (Xia et al., 2019). This may explain why the Fe(III)-coating (dominated by akaganeite) on the pumice surface facilitated the sorption of sulfate from spring water (contributing to sulfate concentrations up to ∼16 mg/L).

Altogether, geochemical changes observed suggest that the Fe(III)-coated pumice deployed into the spring water captured a combination of both Fe(III)-reducers and some sulfate-reducers potentially capable of reducing Fe(III) and sulfate in the presence of added electron donors (lactate and acetate). Similarly, reduction of the relatively low levels of nitrate detected in these experiments, was only coupled to organic matter oxidation in the deployed uncoated/Fe(III)-coated pumice slurries amended with lactate and acetate (Figures 3, 4).

### Fe(III)-Reducers Captured and Enriched by the Deployed and Incubated “Microbe Baits”

At the end of the laboratory incubation period, both the deployed Fe(III)-coated and uncoated pumice slurries unamended with electron donors enriched for organisms predominantly affiliated with the Phylum Betaproteobacteria (19–40% of sequences). However, following the addition of electron donors (lactate and acetate), the deployed Fe(III)-coated pumice slurries largely enriched for Deltaproteobacteria while the deployed uncoated pumice slurries amended with electron donors preferentially enriched for Epsilonproteobacteria and Methanomicrobia. Betaproteobacteria and Deltaproteobacteria are known to contain members capable of utilizing Fe(III) as electron acceptor (Islam et al., 2004; Hery et al., 2015). Epsilonproteobacteria on the other hand, are well known to utilize electron acceptors other than Fe(III), such as nitrate, in anoxic organic-rich environments (Esther et al., 2015; Goris et al., 2016) while Methanomicrobia, are methane-producing archaea which utilize CO_2_ as electron acceptor (Gilmore et al., 2017).

At the Genus level (and focusing on Fe(III)-reducers), the results showed that both the freshly retrieved and incubated Fe(III)-coated and uncoated pumice slurries unamended with electron donors enriched for a close relative to *Rhodoferax ferrireducens* (6.4 and 5.8%, freshly retrieved; 15 and 2%, incubated with no electron donor) (Figure 8). Comparative genome-scale modeling of the competition between *R. ferrireducens* and *Geobacter sulfurreducens* in anoxic subsurface environments predicted that *R. ferrireducens* will outcompete *Geobacter* in environments with low carbon, while *Geobacter* species are expected to predominate if higher concentrations are added (Zhuang et al., 2011). This is consistent with the results in present study, as an organism closely related to *R. ferrireducens* was preferentially enriched in all the pumice samples with no added electron donors.

The dominant enrichment of *Geobacter* sp. in the deployed Fe(III)-coated pumice slurries amended with electron donors is consistent with previous reports that this typical Fe(III)-reducing Genus preferentially utilizes acetate as electron donor for Fe(III) reduction (Lovley, 1993, 1997; Coates et al., 1996; Snoeyenbos-West et al., 2000; Islam et al., 2004; Hery et al., 2010; Esther et al., 2015). In addition, the relative abundance of *Desulfovibrio* species, which are sulfate-reducing bacteria, also able to respire nitrate (Marietou, 2016), suggests that the Genus is most likely responsible for sulfate reduction in the deployed Fe(III)-coated pumice slurries amended with electron donors (Figure 5B). *Desulfovibrio* species also possess the ability to directly reduce Fe(III) (Coleman et al., 1993) and prefer lactate as electron donor (Lentini et al., 2012; Hery et al., 2015) which would be consistent with their enrichment in the deployed Fe(III)-coated pumice slurries amended with electron donors.

### Mineralogical Changes in Deployed and Incubated “Microbe Baits”

Transmission Electron Microscope analysis of the deployed Fe(III)-coated pumice slurries amended with electron donors indicated the presence of spherical nanoparticles with a diameter of 6 nm consistent with the morphological features of magnetite (Galateanu et al., 2015; Figure 6A). These morphological features are similar to the TEM images of biogenic magnetite crystals observed by Cutting et al. (2009), where akaganeite was reduced to magnetite by *Geobacter sulfurreducens* in cultures amended with 10 μm AQDS and 20 mM sodium acetate, and incubated for 6 days. Furthermore, biomagnetite could appear as aggregates of cubic or octahedral structures (Mirabello et al., 2016), similar to the ESEM micrograph obtained in Figure 6B. The EDS spectrum revealed the presence of Fe, O, Si, and Cu (Figure 6C), with the Fe and O consistent with an iron oxide (Dong et al., 2000). The Si peak originated from the pumice itself, as XRD analysis of the coated pumice showed it largely contains Quartz (SiO_2_) and Halite (NaCl) while the Cu peaks originated from the TEM copper grid upon which the slurry droplet was placed during imaging. Analysis of the SAED patterns using the *Image J* software revealed that the diffraction rings (220, 311, 400, and 422) corresponded with the following *d* values: 2.99, 2.60, 2.13, and 1.80A, respectively (Figure 6D), which are consistent with the *d* values for magnetite reported by Haavick et al. (2000), Barber and Scott (2002), Glazyrin et al. (2012), and Galateanu et al. (2015).

## Conclusion

The selective capture of Fe(III)-reducers using Fe(III)-coated pumice was demonstrated in this study. Pumice was coated with akaganeite, a bioavailable Fe(III) oxide mineral, and deployed into a spring water well in the West Midlands, United Kingdom for 2 months. A series of microcosm experiments were carried out on the retrieved pumice samples demonstrating the capture of Fe(III)-reducers and the shift in the microbial community due to the stimulation with lactate and acetate. Fe(II) production correlated with the depletion of DOC and organic acids, indicating microbially-mediated Fe(III) reduction. Some of the enriched Fe(III)-reducers partially oxidized lactate to propionate, *n*-butyrate and acetate. The Fe(III) mineral (akaganeite) coated on the pumice was bioreduced to magnetite, an Fe(II)-containing mineral.

The Fe(III)-coated pumice captured and enriched a higher abundance of Fe(III)-reducing microorganisms than those captured and enriched on the uncoated pumice sample, indicating the role of Fe(III) as a suitable substrate or bait for the target organisms. The deployed Fe(III)-coated pumice slurries unamended with electron donors enriched Betaproteobacteria, dominated by *Rhodoferax ferrireducens*, a typical Fe(III)-reducer known to thrive in anoxic environments with low carbon. This indicates that native organic matter captured by the deployed pumice samples could enrich for DIRB Genera other than *Geobacter* and *Shewanella* species; implying that enrichment of organisms that are better adapted to lower levels of natural organic matter is possible. However, in the deployed Fe(III)-coated pumice slurries stimulated with lactate and acetate, there was a shift in the microbial community, largely enriching Deltaproteobacteria, dominated by *Geobacter* and *Desulfovibrio* species. *Desulfovibrio* are specialist sulfate-reducers; therefore, were most likely enriched by the presence of sulfate and Fe(III) on the pumice materials.

These results suggest that the Fe(III)-coated pumice “*microbe bait*” system tested here can be used to enrich for a wide-range of Fe(III)-reducers. More specifically, the Fe(III)-coated pumice “*microbe baits*” can be used to capture DIRB that cannot outcompete better characterized model organisms such as *Geobacter* sp. in organic-rich media e.g., *Rhodoferax* species, to help better understand the nano-scale interactions between Fe(III)-reducers and insoluble iron minerals (and other contaminants) under conditions more representative of field conditions. Thus the “*microbe bait*” can be used to capture a more complex microbial consortia for *in situ* spatial analysis (alongside native organic matter). The use of these systems is now being focused on other more complex aquifer systems, to help understand better the complex interplay between microbes, Fe, organics and other trace metals, and the subsequent impact on water quality.

## Data Availability Statement

The raw data obtained in this research were deposited to NCBI SRA (Sequence Read Archive; http://www.ncbi.nlm.nih.gov/sra/) under the project accession number: PRJNA603272.

## Author Contributions

BM was the lead researcher, collected samples from the field, conducted the laboratory experiments, analyzed the raw data, interpreted the results, and wrote the manuscript draft. CB conducted microbial analyses and processed the data. JL gave full conceptual guidance and extensively reviewed the manuscript with BD.

## Conflict of Interest

The authors declare that the research was conducted in the absence of any commercial or financial relationships that could be construed as a potential conflict of interest.
