# Supplementary material for: A Novel “Microbial Bait” Technique for Capturing Fe(III)-Reducing Bacteria
Source: Front Microbiol. 2020 Mar 11;11:330. doi: 10.3389/fmicb.2020.00330 (PMC7078115; doi:10.3389/fmicb.2020.00330)
Supplement: Supplementary file 1 [file Data_Sheet_1.docx]

**Supplementary Information**

**
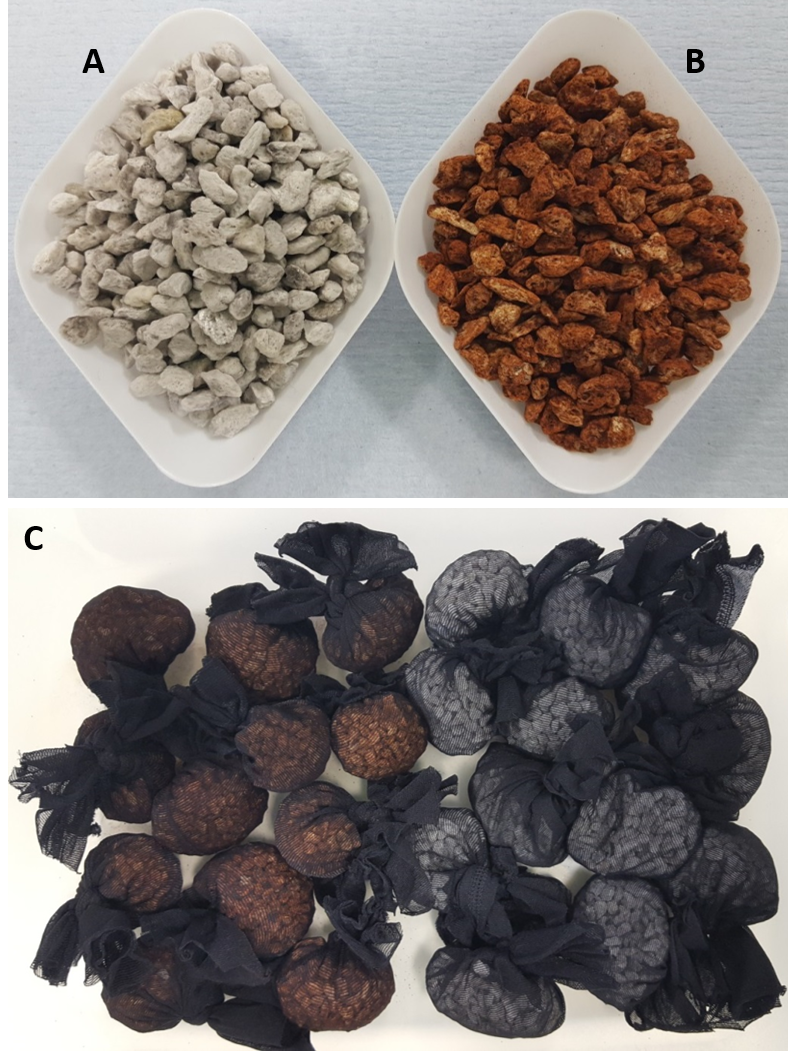
**

**Figure S1: (A)** Uncoated pumice and **(B)** Fe(III)-coated pumice samples prior to deployment, and **(C)** Fe(III)-coated and uncoated pumice samples (10 g each) held in pieces of (improvised) nylon stocking prior to deployment into a Springwater well at West Midlands, UK.

**
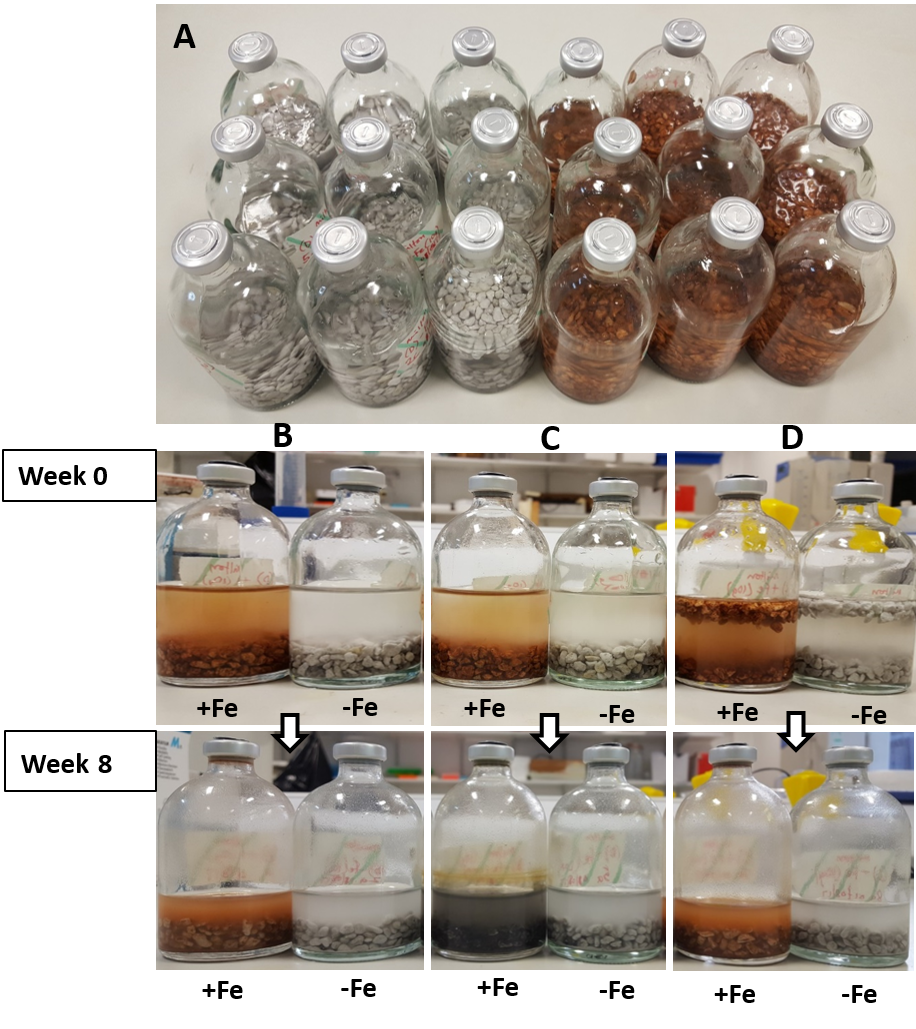
**

**Figure S2: (A)** Uncoated and Fe(III)-coated pumice samples (10 g each) filled into 100 ml serum bottles containing synthetic groundwater (40 ml each) set up in triplicate. **(B)** Deployed Fe(III)-coated and uncoated pumice unamended with electron donors, **(C)** deployed Fe(III)-coated and uncoated pumice amended with lactate and acetate, and **(D)** non-deployed Fe(III)-coated and uncoated pumice unamended with electron donors in 100 ml serum bottles, incubated for 8 weeks at 20℃. + Fe represents Fe(III)-coated pumice; - Fe represents uncoated pumice.

**Figure S3:** X-ray diffractogram of Fe(III) oxide mineral coated on the pumice prior to deployment in the field. Identified as Akaganeite, similar to the observations of Cutting et al. (2009) and Deliyanni and Peleka (2010).


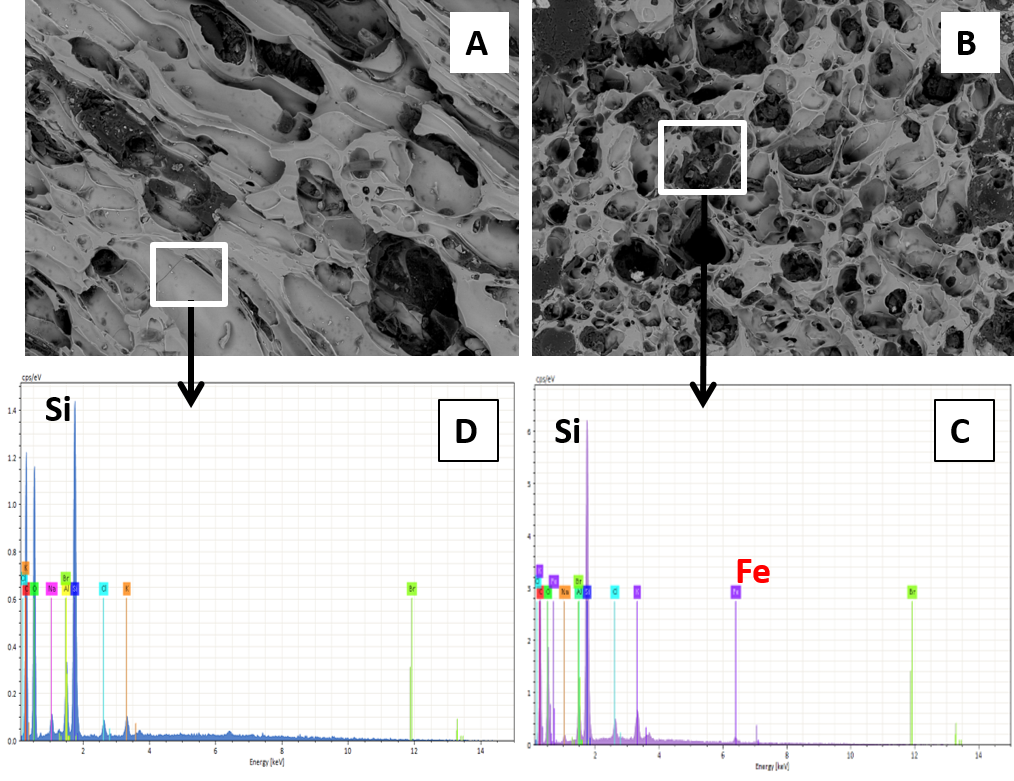


**Figure S4:** ESEM micrographs and EDS spectra of thin-sections of Fe(III)-coated pumice showing the non-coated (**A** and **D**) and Fe(III)-coated (**B** and **C**) interior regions of the rock.

**
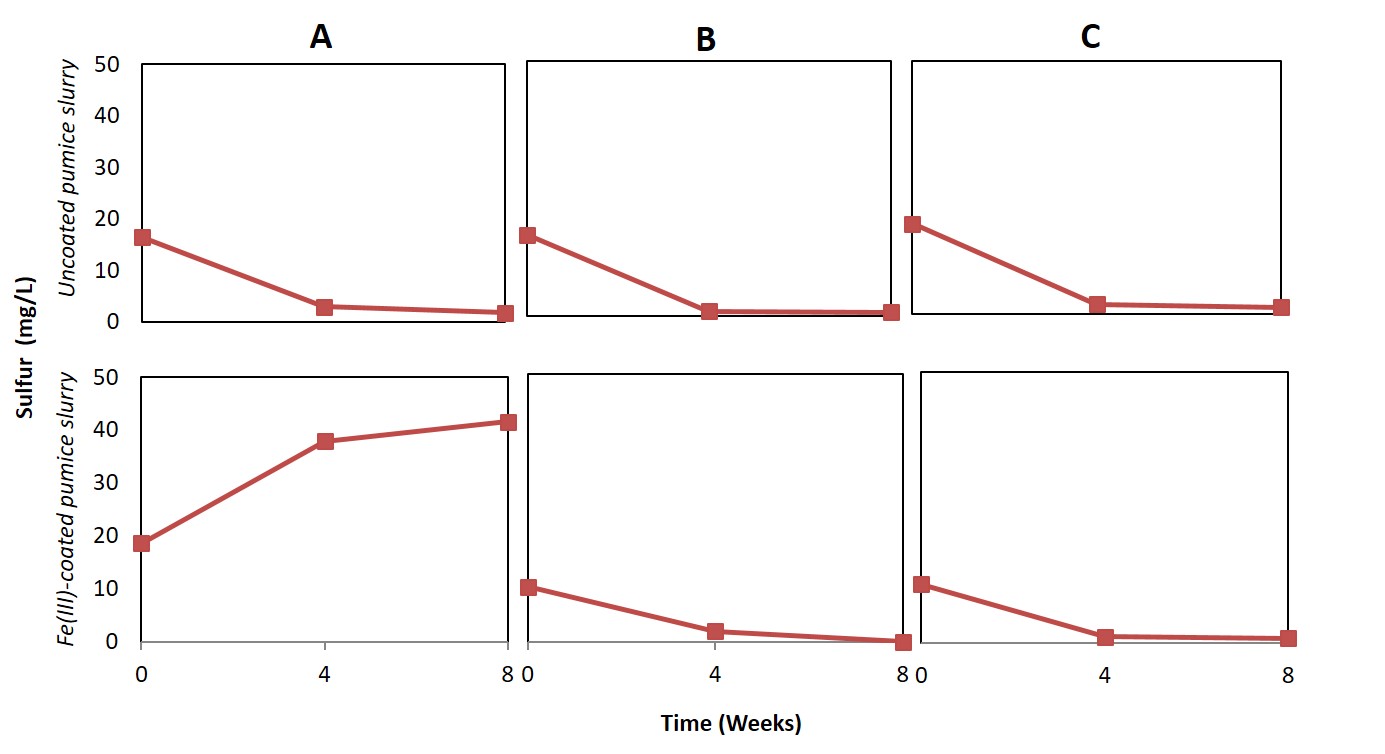
Figure S5:** Sulfur concentrations in uncoated and Fe(III)-coated pumice slurry samples incubated for 8 weeks at 20℃. **(A)** Deployed pumice unamended with electron donors. **(B)** Deployed pumice amended with lactate and acetate. **(C)** Non-deployed pumice unamended with electron donors (Control). Data are the means ± standard errors of triplicates.

**References**

Cutting, R.S., Coker, V.S., Fellowes, J.W., Lloyd, J.R., and Vaughan, D.J., 2009. Mineralogical and morphological constraints on the reduction of Fe (III) minerals by *Geobacter sulfurreducens*. *Geochim. Cosmochim. Acta*, 73(14), 4004-4022.

Deliyanni, E.A. and Peleka, E.N. (2010). Nanohybrid anionic surfactant akaganeite: Preparation, structural analysis and cation sorption. *Int. J. Environ. Technol. Manage*. 12(2-4), 1-14.
